# Limited Mitochondrial Activity Coupled With Strong Expression of CD34, CD90 and EPCR Determines the Functional Fitness of *ex vivo* Expanded Human Hematopoietic Stem Cells

**DOI:** 10.3389/fcell.2020.592348

**Published:** 2020-12-15

**Authors:** Luena Papa, Mansour Djedaini, Tiphaine C. Martin, Mahtab Zangui, Kristin G. Beaumont, Robert Sebra, Ramon Parsons, Christoph Schaniel, Ronald Hoffman

**Affiliations:** ^1^Division of Hematology and Medical Oncology, Tisch Cancer Institute, Icahn School of Medicine at Mount Sinai, New York, NY, United States; ^2^Department of Oncological Sciences, Tisch Cancer Institute, Icahn School of Medicine at Mount Sinai, New York, NY, United States; ^3^Department of Genetics and Genomic Sciences, Icahn School of Medicine at Mount Sinai, New York, NY, United States; ^4^Black Family Stem Cell Institute, Icahn School of Medicine at Mount Sinai, New York, NY, United States; ^5^Mount Sinai Institute for Systems Biomedicine, Icahn School of Medicine at Mount Sinai, New York, NY, United States

**Keywords:** *ex vivo* expansion, valproic acid, phenotype, mitochondrial membrane potential, EPCR, CD90, functional fitness

## Abstract

*Ex vivo* expansion strategies of human hematopoietic stem cell (HSC) grafts with suboptimal stem cell dose have emerged as promising strategies for improving outcomes of HSC transplantation in patients with hematological malignancies. While exposure of HSCs to *ex vivo* cultures expands the number of phenotypically identifiable HSCs, it frequently alters the transcriptomic and metabolic profiles, therefore, compromising their long-term (LT) hematopoietic reconstitution capacity. Within the heterogeneous pool of expanded HSCs, the precise phenotypic, transcriptomic and metabolic profile and thus, the identity of HSCs that confer LT repopulation potential remains poorly described. Utilizing valproic acid (VPA) in *ex vivo* cultures of umbilical cord blood (UCB)-CD34^+^ cells, we demonstrate that expanded HSCs phenotypically marked by expression of the stem cell markers CD34, CD90 and EPCR (CD201) are highly enriched for LT-HSCs. Furthermore, we report that low mitochondrial membrane potential, and, hence, mitochondrial activity distinguishes LT-HSCs within the expanded pool of phenotypically defined HSCs. Remarkably, such reduced mitochondrial activity is restricted to cells with the highest expression levels of CD34, CD90 and EPCR phenotypic markers. Together, our findings reveal that high expression of CD34, CD90 and EPCR in conjunction with low mitochondrial activity is critical for identification of functional LT-HSCs generated within *ex vivo* expansion cultures.

## Introduction

A long-standing goal in the field of hematopoietic stem cells (HSCs) has been the identification and characterization of functional HSCs with long term (LT)-repopulating capacity upon transplantation. LT-HSCs sustain hematopoiesis throughout the lifespan of an individual by constantly replenishing the hematopoietic system with committed progenitors (HPCs) and differentiated blood cells. This LT-repopulating capacity is due to the HSC’s ability to balance self-renewal with commitment decisions (Orkin and Zon, 2008; Seita and Weissman, 2010). Such balance is controlled by complex mechanisms that rely on both the transcriptomic and metabolic properties of LT-HSCs (Jang and Sharkis, 2007; Schieke et al., 2008; Takubo et al., 2013; Warr and Passegue, 2013; Kohli and Passegue, 2014; Maryanovich et al., 2015; Mohrin et al., 2015; Vannini et al., 2016; Anso et al., 2017; Papa et al., 2019b; Spurlock et al., 2019). The transcriptome and metabolism of LT-HSCs are intrinsically coupled to their mitochondrial activity, which is profoundly altered during HSC commitment and maturation. Although the role of the mitochondrial bioenergetic profile during differentiation has been recently challenged, the impact of mitochondrial metabolism and activity in homeostasis and maintenance of primary HSCs with LT-repopulating potential remains undeniable (Anso et al., 2017; de Almeida et al., 2017; Bonora et al., 2018; Ito et al., 2019; Morganti et al., 2019; Liang et al., 2020).

LT-HSCs can restore sustained functional hematopoiesis in patients with blood disorders and refractory hematological malignancies following allogeneic HSC transplantation. Different sources of donor HSCs, including mobilized peripheral blood (PB), bone marrow (BM) and umbilical cord blood (UCBs) stem cells can be used as grafts. However, the limited numbers of HSCs with LT-reconstituting capacity present in a single UCB unit represents a major challenge for the use of UCBs in clinical transplantation settings with adult patients. To overcome this limitation, culture strategies to *ex vivo* expand UCB-HSCs have been pursued for decades (Boitano et al., 2010; Dahlberg et al., 2011; Chaurasia et al., 2014; Fares et al., 2014; Mehta et al., 2015; Huang et al., 2019; Papa et al., 2020a). Despite significant progress, the identity of HSCs that confer LT-repopulating potential following *ex vivo* HSC expansions remains elusive (Psatha et al., 2017; Chen et al., 2019). While numerous phenotypic surface markers are used to enrich for HSC subpopulations, the true identity and the precise phenotypic, transcriptomic and metabolic profile of *ex vivo* expanded human HSCs with LT-repopulating capacity remains poorly described. Indeed, the functional identity of *ex vivo* expanded HSCs has been reported to be discordant with HSC phenotype. This is in part due to the great heterogeneity of HSCs. Moreover, exposure of HSCs to *ex vivo* culture conditions compromises the metabolic and transcriptomic properties of LT-HSCs in spite of the expansion in the number of phenotypically identifiable HSCs. Although abundant in numbers, such *ex vivo*-expanded cells with an HSC phenotype, in the past, have been frequently incapable of sustaining long-term post-transplant hematopoietic reconstitution. In fact, the discordance between the phenotype and LT-functional properties of expanded HSCs has often compromised the success of many HSC expansion approaches.

Recently, several *ex vivo* expansion strategies, including those based on treatment with stemreginin 1 (SR1), the pyrimidole derivative UM171, nicotinamide (NAM), and valproic acid (VPA) have shown promising results not only in pre-clinical studies but also in ongoing clinical trials (Boitano et al., 2010; Peled et al., 2012; Chaurasia et al., 2014; Fares et al., 2014; Wagner et al., 2016; Horwitz et al., 2019; Islam and Horwitz, 2019; Cohen et al., 2020). In addition to being safe, these *ex vivo*-expanded grafts have led to rapid and sustained multilineage hematopoietic reconstitution in allogeneic recipients. Initial data from our clinical trial using VPA-expanded allogeneic UCB grafts (NCT03885947) have shown persistent (more than 1 year) multilineage donor cell reconstitution with especially rapid T cell and platelet engraftment.

We previously reported that VPA treatment results in *ex vivo* expansion of human HSCs from UCB-CD34^+^ cells. This expansion is accompanied by the acquisition and retention of a transcriptomic and metabolic profile that closely resembles that of human and non-human primary (unmanipulated) LT-HSCs (Chaurasia et al., 2014; Iancu-Rubin and Hoffman, 2015; Papa et al., 2018). Moreover, VPA-expanded HSCs exhibit a remodeled primitive mitochondrial network that is characterized by limited levels of reactive oxygen species (ROS) and mitochondrial activity. Indeed, HSCs expanded with VPA displayed an enriched glycolytic profile and reduced mitochondrial oxidative phosphorylation (OXPHOS) activity. Remarkably, human VPA-expanded HSCs established LT-hematopoietic reconstitution following serial transplantation into sublethally irradiated murine recipients (Chaurasia et al., 2014; Papa et al., 2020a).

Here we provide a comprehensive characterization of the phenotypic properties, mitochondrial fitness and activity, and functional capacity of *ex vivo*-VPA expanded HSCs using classical and novel approaches that include both bulk and single cell transcriptomic, flow cytometric and single cell imaging analyses alongside *in vitro* functional assays. We found that within the human expanded grafts capable of establishing LT-hematopoiesis, HSCs of the CD34^+^CD90^+^EPCR^+^CD38^–^CD45RA^–^ phenotype possess the key properties that define LT-HSCs. We also report that low mitochondrial membrane potential (MMP) and, hence, mitochondrial activity distinguishes the LT-HSCs within the expanded pool of phenotypically defined HSCs. Remarkably, such low MMP is tightly coupled to high expression levels of the HSC phenotypic markers CD34, CD90 and EPCR. Our study suggests that both robust expression of stem cell associated surface markers and low mitochondrial activity should be used as critical properties to identify human HSCs with LT-regenerative potential in *ex vivo* expansion cultures.

## Materials and Methods

### CD34^+^ Cells

Fresh UCBs were purchased from the Placental Blood Program at the New York Blood Center. Mononuclear cells were isolated from UCBs by Ficoll-Hypaque (GE Healthcare) density centrifugation, followed by CD34^+^ cell selection by immunomagnetic sorting using the CD34 Microbead kit (Miltenyi) and the AutoMACS Pro Separator (Miltenyi) as previously described (Papa et al., 2019a). Cryopreserved CD34^+^ cells from G-CSF mobilized peripheral blood (PB) or bone marrow (BM) aspirates harvested from healthy adult donors were purchased from AllCells.

### *Ex vivo* Expansion

CD34^+^ cells were cultured in StemSpan SFEM II (Stemcell Technologies) supplemented with 150 ng/mL human stem cell factor (SCF), 100 ng/mL human fms-like tyrosine kinase receptor 3 (FLT-3-ligand), 100 ng/mL human thrombopoietin (TPO), and 50 ng/mL human interleukin 3 (IL3) (R&D Systems). Cells were seeded at 3.3 × 10^4^ cells/mL in 22.1 mm wells (12-well plates) and incubated in a humidified incubator maintained at 37°C with 5% CO2. Cells were incubated with the cytokine cocktail alone for 16 h (cytokine priming) followed by addition of VPA at 1 mM (Sigma-Aldrich) for 7 additional days.

### Phenotypic Assessment by Flow Cytometry and FACS Sorting

Unmanipulated UCB, BM or mobilized PB CD34^+^ cells or cells from cultures treated with cytokines alone or cultures treated with VPA were stained with anti-human CD34-APC (BD Pharmingen), CD90-FITC (Thermo Fisher Scientific), CD201-PE (Bio-Legend), CD45RAef-506 (Thermo Fisher Scientific), CD38 SB-702 (Thermo Fisher Scientific) for 30 min at 4°C, washed and analyzed using an Attune flow cytometer (Thermo Fisher Scientific). Analyses were performed with Attune software (Thermo Fisher Scientific) and FlowJo Software (BD Biosciences). Compensation parameters are assessed by using AbC^TM^ Total Antibody Compensation Bead Kit (Thermo Fisher Scientific) according to the manufacturer instructions. Doublet exclusion was performed by FSC-H/FSC-A selection followed by SSC-H/SSC-A selection. Gating of positive/negative population was performed by using fluorescence minus one control (FMO) strategy (Papa et al., 2020b). Purification of the various CD34^+^ cell subpopulations is performed in media supplemented with cytokines or cytokines and VPA using a BD FACSAria (BD Bioscience).

### Evaluation of Mitochondrial Membrane Potential (MMP)

Mitochondrial membrane potential was measured using tetramethylrhodamine methyl ester (TMRM) (Invitrogen) in the presence of the efflux pump inhibitor, verapamil (Sigma-Aldrich). Cells were incubated for 20 min at 37°C with 200 nM TMRM, 50 μM verapamil and anti-human CD34 PE-Cy7 (Thermo Fisher Scientific), CD90 APC-Cy7 (Thermo Fisher Scientific) and CD201 APC (BioLegend) antibodies. Following 2 washes with 1xPBS supplemented with 2% FBS, the cells were analyzed immediately using an Attune flow cytometer (Thermo Fisher Scientific) or an Amnis ImageStreamX Mk II Imaging Flow Cytometer (Luminex). Sorting and analyses of TMRM stained cells was performed using compensation parameters determined by using the AbC^TM^ Total Antibody Compensation Bead Kit (Thermo Fisher Scientific) according to the manufacturer’s instruction. For TMRM data analyses, doublet exclusion was performed by FSC-H/FSC-A selection followed by SSC-H/SSC-A selection. Gating of positive/negative populations were performed using the FMO staining method (Papa et al., 2020b). Image acquisition and analysis on ImageStreamX was performed after doublet exclusion as previously described https://onlinelibrary.wiley.com/doi/full/10.1002/cyto.a.23064. Images were acquired with the 40× magnitude objective using INSPIRE^®^ software. Analysis was done using IDEAS^®^ software. Cells were gated using the Area versus Aspect Ratio scatterplot, which is the ratio between the minor and major axis of the object image fitting ellipse and is commonly used for identification of cells. Using the standard setting mask (MC_mask), intensity for each fluorescent staining per cell was quantified and plotted after selection of High and Low MC_intensity for the TMRM.

### Cell Loading and Culture in Nanofluidic Chip Wells

Single cell growth and cell surface marker expression analysis were carried out on nanofluidic chips equipped with Beacon OptoSelect technology, version 2001 (SSRLx), designed by Berkeley Lights Inc. (BLI, Emeryville, CA, United States). For all cell cultures, the BLI chips were primed using media buffered with 5% CO_2_. We used, for the control condition, media containing the cytokine cocktail alone, while for the VPA condition, media contained the combination of the cytokine cocktail plus VPA. Cells were obtained at 3.3 × 10^4^ cells/mL in the desired media, loaded onto a BLI chip at 25°C, and penned randomly as single cells using Opto-Electronic Positioning (OEP) force was produced by a waveform generator with peak-to-peak voltage of 1.1 V, at a velocity of 5 μm/s. After loading, the BLI chips were flushed with fresh media and cells were cultured at 37°C in 5% CO_2_-buffered conditioned media, perfusing at 0.01 μL/s. Cells were cultured on the BLI chips for 7 days, with image acquisition every 2 h. At days 0, 2, 4, and 6, cells were stained using an anti-CD34 antibody, followed by incubation with secondary antibody for signal amplification, and TMRM. Specifically, media containing anti-CD34 (BD Bioscience) at 1/200 dilution and TMRM at 200 nM was loaded onto the chip and incubated for 90 min, followed by 90 min incubation with Alexafluor 488-conjugated goat anti-mouse IgG (Thermo Fisher Scientific) at 20 μg/mL. After staining, the BLI chips were flushed with fresh media and perfusion/incubation was resumed as described above. Images were acquired every 2 h in brightfield, FITC and Texas Red channels. Control samples were imaged with exposure times of 1.5 and 3 s for FITC and Texas Red, respectively, and VPA samples were imaged with exposure times of 1 and 2 s for FITC and Texas Red, respectively.

### Image Processing and Analysis of Cells in the BLI Platforms

Background subtraction of fluorescence images was performed using Fiji software by mathematically subtracting the average intensity within a conserved background region of interest. Cell counting was done using an automated edge detection algorithm followed by visual confirmation of detected cells in brightfield and FITC/Texas Red images. Measurements of fluorescent intensity were performed in Fiji using the “Measure” function with a multi-point selection of all individual cells in each pen.

### Limiting Dilution Long-Term Culture Initiating Cell (LTC-IC) Assay

The LTC-IC assay, a surrogate *in vitro* assay for human HSC functionality, was performed as previously described (Weaver et al., 1997). The number of LTC-ICs in a particular cell population was determined by limiting dilution analysis. Briefly, sorted cells were seeded on irradiated AFT024 feeder cells (Moore et al., 1997; Thiemann et al., 1998; Nolta et al., 2002) at 500, 250, 125,62,31,15,7 and 3 cells per well of a 96 well plate through a serial dilution. Cultures were incubated in a humidified atmosphere containing 5% CO_2_ in air at 37°C. Culture plates were fed with half medium changes on a weekly basis for a total of 7 weeks. LTC-IC frequency was calculated by scoring cobblestone area forming cells and applying Poisson distribution statistic and linear regression polynomial curve fitting. Calculations were done using the limiting dilution software at http://bioinf.wehi.edu.au/software/elda/ as previously described (Hu and Smyth, 2009).

### Cell Isolation for Single-Cell and Bulk RNA-Seq

Viable CD34^+^ from either unmanipulated UCBs (Day 0) or from cultures containing Stemline II media (Millipore Sigma) supplemented with VPA or cytokines alone were FACS-purified and subjected to either single cell or bulk RNA-seq.

### Single-Cell RNA-Seq and Data Analysis

10x Genomics Chromium Single Cell Gene Expression Solution, a droplet-based ultra-high-throughput single-cell (sc) RNA-seq system, was used to sequence the transcriptome of single cells from Day 0 and 2 cultures. scRNA-seq data generated about 166 million reads per sample. A total of 1,525 cells at Day 0 and 5,002 cells at Day 2 were identified and analyzed. The primary data were processed using the Cell Ranger pipeline v1.3 (Zheng et al., 2017). The Cell Ranger pipeline aligned 70–80% of the reads uniquely to the human reference genome hg19. Analysis of scRNA-seq data was then performed using R libraries available in R version 4.0.0. Two single-cell datasets (Day 0 and Day 2) were read using the R library DropletUtils and merged using R library scater. Empty drops were removed. The number of cells with high percentage of mitochondrial reads, which is a proxy for cell damage, was evaluated. However, all cells including those with a high percent of mitochondrial reads were kept as this could be an effect of VPA treatment (90 cells for Day 0 and 131 cells for Day 2). The two datasets were normalized based on common genes using R library batchelor and 9 clusters were identified using the R function buildSNNGraph of R library scran and the R function cluster_louvain of R library igraph. We scored each cluster of cells expanded with VPA for 2 days using their average gene expression against a gene set at a single-sample level (gene sets for Long-term HSCs, Short-term HSCs, early, late and intermediate progenitors) (Ivanova et al., 2002) using R singscore library (V1.8.0) and visualized different scoring in heatmap. Heatmap was drawn using R ComplexHeatmap library (v2.0.0).

### Visualization of Single Cells

The Uniform Manifold Approximation and Projection (UMAP) dimensional reduction technique for single cells was run and single cells were visualized in the two first UMAP dimension components using R library scater (McCarthy et al., 2017). Depending on the figure, cells were color-coded based on their cluster labels or the level of expression of the gene of interest. To help with visualization of cells with low expression of the gene of interest, cells that had no mapped reads of the gene of interest were excluded.

### Bulk RNA-Seq Data Analysis

Bulk RNA-seq data generated about 44–81 million single-end 1 × 100 reads per sample. The quality of single-end reads with FASTQC (version 0.11.8) was first evaluated to remove adapters, known artifacts, and then quality trimmed (PHRED quality score < 10) reads with BBDUK from BBTOOLS (version 37.53) were filtered. Reads that became too short after trimming (*N* < 60 bp) were discarded. Transcript-level quantification of cleaned mRNA-seq data was estimated using SALMON (version 1.0.0) by a quasi-mapping on the human reference gene annotation GDC.h38 GENCODE v22. Analysis of bulk mRNA-seq data was performed using R libraries available in R version 4.0.0. Differential gene expression between isolated UCB-CD34^+^ cells cultured with cytokines and isolated UCB-CD34^+^ cells cultured with VPA for day 2, 4, and 6, respectively, were performed at gene level (Ngene = 59,979) using R tximport library (version 1.12.3) on R (version 3.6.0). We also performed differential gene expression analysis between unmanipulated UCB-CD34^+^ cells (Day 0), the isolated UCB-CD34^+^ cells from cultures treated with cytokines alone as well as from the cultures treated with VPA for Day 2, 4, and 6, respectively. We pre-filtered genes and eliminated all genes that had <10 reads in total (Ngene = 29,772), and assessed differential gene expression on the remaining genes using R DESeq2 library (version 1.24.1). Differentially expressed genes between two conditions were scored when the adjusted p-value was at FDR 5% and log2 fold change was >2.

### Visualization of Bulk RNA-Seq Data

We visualized the differentially expressed genes using Volcano plot and highlighted three genes of interested (PROCR/EPCR/CD201), THY1/CD90, ITGA6/CD49f), using R Enhanced Volcano library (version 1.2.0). To visualize the change of gene expression of the genes of interests at all the tested time points of *ex vivo* cultures, we visualized the mean and standard deviation of gene expression at each time points (day 0, 2, 4, and 6) and for each culture condition.

### Statistical Analysis

Values are shown as mean ± SEM. Statistical analysis were performed by descriptive statistics such as median and minimum-maximum values. Generalized linear mixed models were used to evaluate the differences. Negative-binomial and Beta models were fitted for counts and percentages of various subpopulations of HSCs, respectively. The analyses were conducted using the statistical software R version 4.0.0 (R Core Team, 2020). Other statistical analyses were performed using GraphPad Prism 7.0 software. *P*-values of less than 0.05 were considered to indicate statistical significance. Student’s *t* test was used for comparisons between two groups, whereas two-way ANOVA were used for comparisons among multiple groups and *P*-values.

## Results

### VPA Generates *ex vivo* Phenotypically Defined LT-HSCs

**FIGURE 1:**
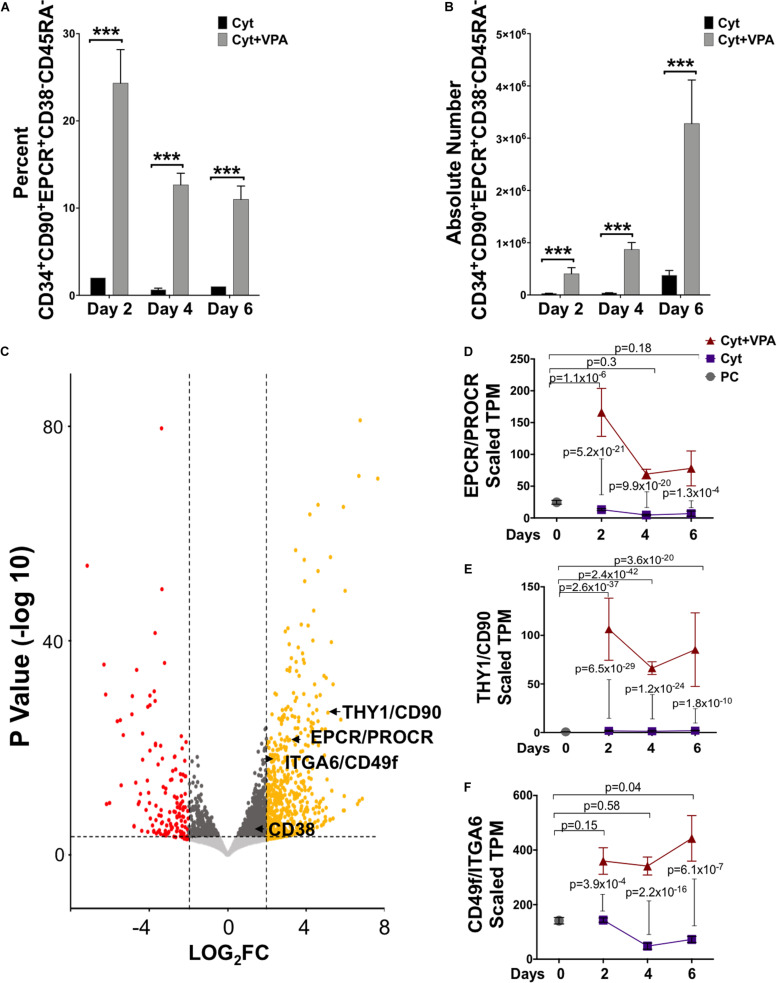
VPA treatment generates phenotypically identifiable LT-HSCs in *ex vivo* cultures. **(A,B)** Percentage **(A)** and absolute number **(B)** of viable CD34^+^CD90^+^EPCR^+^CD38^–^CD45RA^–^ cells generated throughout 6 days of culture of UCB-CD34^+^cells with either cytokines alone or in combination with VPA (Cyt+VPA) as measured by acridine orange/propidium iodide staining and assessed by flow cytometry analysis (*n* = 3). ***p < 0.001 as determined by Negative-binomial and Beta models fitted for counts and percentages, respectively. **(C)** Volcano plot indicating differentially expressed genes between CD34^+^ cells isolated from the 4-day cultures treated with VPA or cytokines alone and analyzed by bulk RNA-seq (*n* = 3). A total of 31,703 genes with a fold-change (FC) cut off of 2 and *p*-value cutoff of 8 × 10^–4^ were characterized. Red dots represent significantly down-regulated genes with a Log_2_ fold change > 2 in VPA-CD34^+^cell cultures compared to those in cultured cells treated with cytokines alone. Yellow dots represent significantly up-regulated genes with a Log_2_ fold change > 2 in VPA-cultures compared to those cultured with cytokines alone. The transcripts for HSC phenotypic markers EPCR, CD90 and CD49f are highlighted. **(D–F)** Gene expression (TPM, transcript per million) of EPCR **(D),** CD90 **(E)**, and CD49f **(F)** extracted from bulk RNA-seq analysis of the uncultured UCB-CD34^+^ cells (PC; gray circle), CD34^+^cells isolated from cultures expanded with cytokines alone (Cyt; purple square) or VPA (Cyt+VPA, red triangle) for the indicated time points. *P*-values in **(D–F)** represent adjusted *p*-values of differential gene expression. N indicates number or experiments.

We previously reported that combination treatment with a cytokine cocktail and VPA (referred hereafter as VPA) leads to generation of a pool of HSCs that are capable of establishing LT-hematopoietic reconstitution in mice (Chaurasia et al., 2014), and likely in human patients (unpublished data). Remarkably, VPA-expanded HSCs possess a phenotypic, transcriptomic and mitochondrial/metabolic profile that closely resembles that of primary (unmanipulated) LT-HSCs (Papa et al., 2018). This pool of VPA-expanded HSCs is highly enriched for cells expressing phenotypic markers reported to mark unmanipulated LT-HSCs, including CD90 and CD49f. However, expression of these phenotypic markers does not reliably define manipulated and expanded HSCs with LT functionality in *ex vivo* cultures (Psatha et al., 2017; Chen et al., 2019). Thus, to precisely determine the phenotype of *ex vivo* expanded HSCs in cultures initiated with UCB-CD34^+^ cells, we further assessed expression of the cell surface marker EPCR combined with CD38 and CD45RA. EPCR is a relatively newly identified phenotypic marker reported to identify human LT-HSCs not only in unmanipulated UCBs but notably, in UM171 *ex vivo* expanded UCB-CD34^+^ cell cultures (Fares et al., 2017). By contrast, expression of both CD38 and CD45RA is negatively associated with human HSC function (Majeti et al., 2007). Flow cytometric analysis revealed that during the entire period of culture, VPA treatment compared to cytokines alone significantly increased both the percentage and the absolute number of HSCs phenotypically defined as CD34^+^CD90^+^EPCR^+^CD38^–^CD45RA^–^ (Figures 1A,B), or CD34^+^CD90^+^CD38^–^CD45RA^–^ (Supplementary Figures 1A,B). Interestingly, about 40% of VPA-expanded CD34^+^CD90^+^CD38^–^CD45RA^–^ cells expressed EPCR. The significant increase in the percentage of phenotypically identifiable HSCs with the CD34^+^CD90^+^EPCR^+^CD38^–^ CD45RA^–^ phenotype occurred as early as 2 days following exposure of UCB-CD34^+^ cells to VPA (Figure 1A). This increased percentage, which was not evident in cultures treated with the cytokine cocktail alone, was accompanied by a very modest increase in the absolute number of CD34^+^CD90^+^EPCR^+^CD38^–^CD45RA^–^ cells (Figure 1B). More prolonged incubation with VPA (days 4–6), however, resulted in a twofold decrease in the percentage of CD34^+^CD90^+^EPCR^+^CD38^–^CD45RA^–^ cells as compared to that observed on day 2 (Figure 1A). In spite of this decline, the absolute number of these phenotypically defined HSCs was increased by fourfold (Figure 1B). These data are consistent with our previous report that indicated an increase in the absolute number of HSCs marked by CD34, CD90, and CD49f expression between days 4–6 of culture as opposed to 2-day treatment with VPA. Such an increase was a consequence of the greater number of cell divisions (Papa et al., 2018).

To examine whether VPA treatment induced an enrichment in transcripts encoding for HSC surface markers, we performed bulk RNA-sequencing and compared the transcriptome of VPA-treated CD34^+^ cells with that of cytokine-treated CD34^+^ cells. This analysis revealed that the pool of VPA-expanded cells possesses a transcriptomic profile that is distinct from the transcriptome of CD34^+^ cells present in cytokine cultures at day 4 (Figure 1C) as well as day 2 and 6 (Supplementary Figures 2A,B). In addition to CD90 and CD49f (Papa et al., 2018), EPCR was found to be significantly and highly upregulated by VPA at all time points tested. In fact, VPA treatment for 2 days resulted in an increase of more than twelvefold (*p*_adj_ = 5.2 × 10^–21^) in transcript levels of EPCR when compared to those expressed by CD34^+^ cells present in cytokine cultures (Figures 1C,D). We also examined the expression dynamics of EPCR transcripts over the entire course of *ex vivo* culture and found that EPCR levels were persistently retained at greater levels in VPA expanded CD34^+^ cells as opposed to CD34^+^ cells treated with cytokines alone (Figure 1D). Consistent with EPCR, CD90 and CD49f transcripts were also significantly induced and remained high in CD34^+^ cells generated in cultures treated with VPA at all tested time points of *ex vivo* cultures (Figures 1E,F). Remarkably, a significant induction in the transcript levels of EPCR, CD90, and CD49f was also evident in VPA-expanded CD34^+^ cells when compared to unmanipulated UCB-CD34^+^ cells (Figures 1D–F). Within the first 2 days of treatment, VPA induced EPCR expression by more than sixfold (*p*_adj_ = 1.14 × 10^–06^). Consistent with the increased expression of HSC phenotypic markers, VPA-expanded cells displayed enrichment in their glycolytic profile (Supplementary Figure 2C). Specifically, genes encoding key regulatory enzymes of the glycolytic pathway were significantly and highly upregulated in VPA-expanded cells. We noted a significant increase in transcripts of ENO2, SLC2A3, HK1 as well as MEIS1A and PBX1 genes. These genes are all highly implicated in glycolytic metabolism and self-renewal of HSCs (Simsek et al., 2010; Kocabas et al., 2015; Gu et al., 2016; Snyder et al., 2018; Wierenga et al., 2019). Thus, these findings indicate that, despite the stress that is often imposed by *ex vivo* cultures, VPA treatment not only maintained but also induced expression of phenotypic markers and glycolytic hallmarks that characterize unmanipulated HSCs with LT-repopulating potential.

### VPA Treatment Induces Rapid and Robust Expression of HSC Phenotypic Markers at the Single Cell Level

**FIGURE 2:**
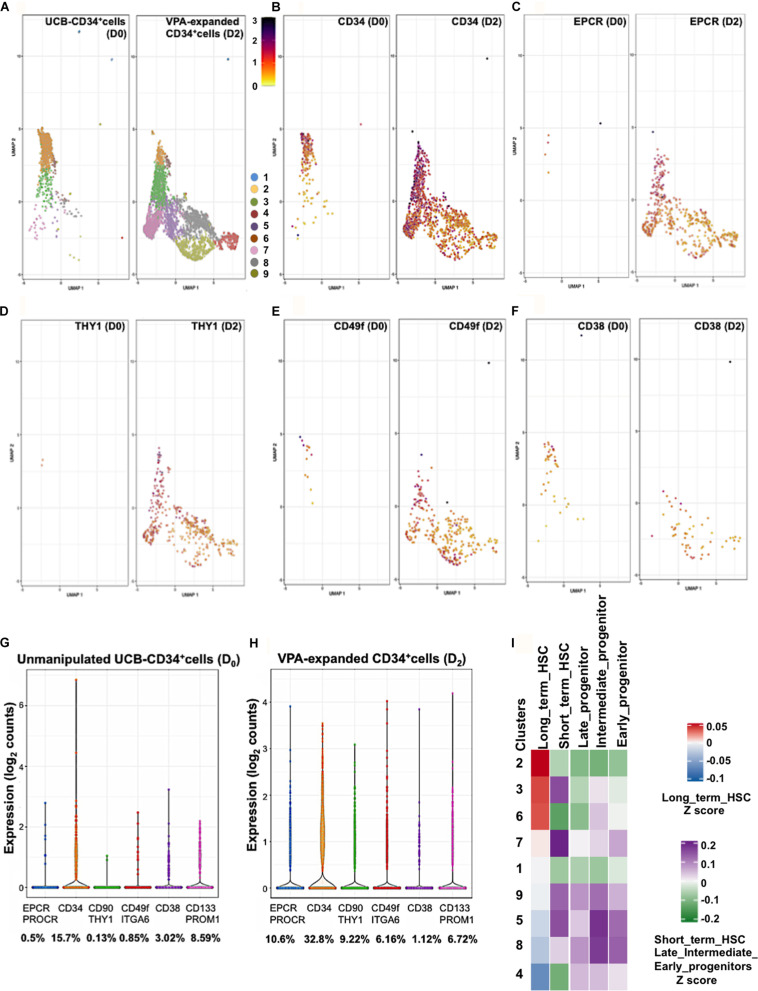
VPA treatment induces transcription of the LT-HSC phenotypic markers. **(A)** GEM Drop-seq analysis of unmanipulated single UCB-CD34^+^ (D0) and CD34^+^ cells isolated from cultures treated with VPA for 2 days (D2). Single unmanipulated UCB-CD34^+^ cells as well as VPA-treated CD34^+^ cells cluster into 9 distinct transcriptional populations by uniform manifold approximation and projection (UMAP) analysis. Each cluster is denoted by a different color. **(B–F)** UMAP projections show expression of CD34 **(B)**, EPCR/CD201 **(C)**, CD90/THY1 **(D)**, CD49f/ITAG6 **(E)**, and CD38 **(F)** genes in single cells. Gene expression levels range from low (yellow) to red (medium) to dark blue (high). Cells that lack expression of the indicated genes were removed from each respective UMAP. **(G,H)** Violin plots representing the transcript levels of each of the indicated genes in single CD34^+^ cells isolated from unmanipulated UCB or from cultures treated with VPA for 2 days. Numbers represent the percentage of cells expressing the indicated transcripts. **(I)** Heatmap-based correlation of gene sets specific to each cluster of VPA-expanded CD34^+^cells for 2 days to gene sets of the LT-term, short-term HSCs and early, intermediate and late progenitors from Ivanova et al. (2002).

Single-cell transcriptomic (scRNAseq) analysis unmasks heterogeneity and provides high-resolution mapping of highly complex systems including hematopoiesis (Lunger et al., 2017; Nam et al., 2019; Pellin et al., 2019). We previously reported that VPA-induced expression of CD90 and CD49f at single cell level (Papa et al., 2018). To assess expression of EPCR at single cell resolution we analyzed scRNAseq data of 1,525 unmanipulated UCB-CD34^+^ cells and 5,002 CD34^+^ cells isolated from cultures treated with VPA for 2 days. These analyses revealed a transcriptionally heterogeneous pool of immunophenotypically defined CD34^+^ cells. Both unmanipulated and VPA treated CD34^+^ cells consisted of the same defined clusters of cells that differed transcriptionally (Figure 2A). However, VPA treatment appears to affect the size of each of these cell clusters at various degrees. This finding strongly indicates that such heterogeneity is not solely the consequence of VPA treatment but rather that the pool of unmanipulated UCB-CD34^+^ cells is already highly heterogeneous. VPA treatment resulted in a significant increase in both the percentage of cells expressing transcripts for CD34 (Figure 2B), EPCR/CD201 (Figure 2C), THY1/CD90 (Figure 2D), and CD49f/ITGA6 (Figure 2E) and their levels. Specifically, compared to unmanipulated UCB-CD34^+^ cells, VPA treatment increased the percentage of cells expressing EPCR by 21-fold, CD34 by 2-fold, CD90 by 70-fold, and CD49f by 7-fold, whereas it decreased the percentage of cells expressing CD38 transcripts by 3-fold (Figures 2G,H). We did not observe a substantial change in the percentage of cells expressing CD133/PROM1 (not shown), which is another phenotypic marker used to identify HSCs (Takahashi et al., 2014). Notably, at single cell resolution, both EPCR and CD90 emerged as genes whose expression was most strongly induced by VPA (Figures 2G,H). In VPA-generated cells, EPCR and CD90 expression levels range from 0.43 to 3.85 reads/cell and 0.38 to 3.08 reads/cell as compared to unmanipulated UCB-CD34^+^cells with values that ranged from 0.79 to 2.63 reads/cell and 0.94 to 0.98 reads/cell, respectively. Consistent with our bulk RNA-seq data, these single cell data indicate that VPA expands a pool of HSCs that are characterized by increased expression of EPCR transcripts as well as transcripts of other phenotypic markers that define LT-HSCs. Next, we interrogated the scRNA-Seq data and compared the signatures of each cluster of VPA-expanded CD34^+^ cells to signatures of various human HSC (LT-HSCs and short-term HSCs) and progenitor subsets (early, intermediate and late progenitors) (Ivanova et al., 2002). This analysis unveiled a strong similarity between the signature of LT-HSCs and those of cells in clusters 2, 3, and 6 (Figure 2I). The other clusters were enriched for cells with signatures that resemble those of committed progenitors including early, intermediate, and late progenitor cells. Moreover, clusters 2, 3, and 6 exhibited a profile characterized overall by lower expression levels of genes that regulate electron transport chain (ETC) function, including MT-ND2, MT-ATP6, NDUFB9, COX8A (Supplementary Figure 3). Notably, compared to all the other clusters, clusters 2, 3 and 6 although heterogeneous are highly enriched for cells that express the greatest levels of CD34, CD90, EPCR transcripts and do not express CD38 transcripts (Figures 2B–F). Thus, cells expanded by VPA marked by the strongest expression of HSC phenotypic markers display signatures that resemble those that characterize human unmanipulated LT-HSCs.

### VPA-Generated HSCs That Are Characterized by Increased Expression of HSC Phenotypic Markers Exhibit Low MMP

**FIGURE 3:**
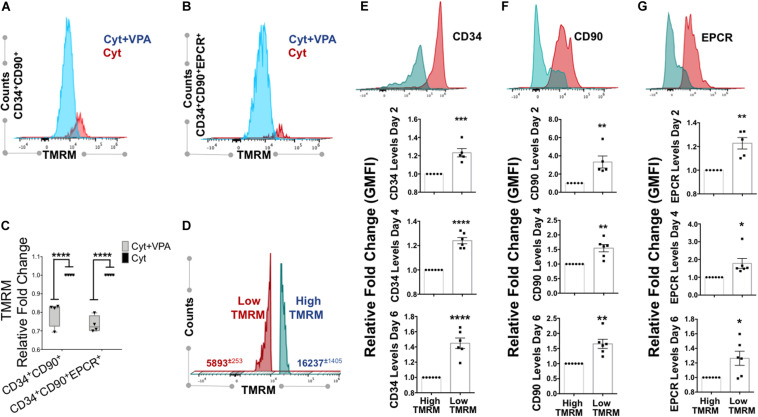
VPA-generated cells with low MMP possess strong expression of HSC phenotypic markers. **(A,B)** Representative flow cytometry plots of MMP as measured by Tetramethylrhodaminemethyl ester (TMRM) retention in the presence of dye inhibitor verapamil in CD34^+^CD90^+^ and CD34^+^CD90^+^EPCR^+^ cells, respectively, generated in cultures treated with cytokines alone (Cyt) and VPA (Cyt+VPA) for 4 days. **(C)** Relative fold change in median fluorescence intensity (MFI) of TMRM staining (MMP) in the indicated subpopulation of cells generated in VPA cultures for 4 days and measured as described in **(A,B)** (*n* = 4). **(D)** Representative flow cytometry plot of viable cells in cultures treated with VPA for 6 days and stained with TMRM in the presence of verapamil. Numbers represent the average of geometric median florescence intensity (GMFI) of cells that retain low TMRM staining (red) and cells that retain high TMRM staining (blue). **(E–G)** Representative flow cytometry plot of CD34 **(E)**, CD90 **(F)**, and EPCR **(G)** expression (top panels) in low and high MMP cells generated in cultures treated with VPA for 6 days and determined by TMRM retention in **(D)**. Lower panels in **(B–D)** represent relative fold changes in respective GMFI of CD34 **(E)**, CD90 **(F)**, and EPCR **(G)** expression in TMRM-high (MMP-high) relative to TMRM low (MMP-low) cells generated in cultures treated with VPA for 2, 4, and 6 days, respectively and stained as described in **(A)** (*n* = 5 for day 2 and *n* = 6 for days 4 and 6). Error bars for **(C)**, SEM; ****p ≤ 0.0001 as determined by two-way ANOVA (Sidak’ multiple comparison test). Error bars for **(E–G)**, SEM; *p ≤ 0.05; **p ≤ 0.01; ***p ≤ 0.001; ****p ≤ 0.0001 as determined by unpaired *t*-test (two-tailed).

Mitochondrial activity is a critical determinant of primary HSCs with LT-hematopoietic reconstituting potential (Vannini et al., 2016; Anso et al., 2017; Spurlock et al., 2019; Liang et al., 2020). We previously demonstrated that VPA-expanded HSCs exhibited a remodeled primitive mitochondrial network characterized by low mitochondrial ROS, mass and MMP (Papa et al., 2018). As dye staining can be affected by xenobiotic efflux (de Almeida et al., 2017; Morganti et al., 2019), to accurately measure MMP in VPA-expanded HSCs we utilized the fluorescent dye reporter TMRM in the presence of verapamil, an inhibitor of dye efflux. Consistent with our published evidence (Papa et al., 2018), VPA-expanded CD34^+^CD90^+^ cells exhibited reduced MMP that was manifested by a low degree of TMRM accumulation even in the presence of verapamil (Figures 3A,C). Specifically, *ex vivo* VPA treated CD34^+^CD90^+^ cells displayed an MMP that was 20–30% lower than that of the same subpopulation of cells present in cultures treated with cytokine cocktail alone (Figure 3C). Similarly, in VPA treated cultures, expanded HSCs phenotypically identified as CD34^+^CD90^+^EPCR^+^ cells also possessed significantly lower MMP when compared to the same subpopulation of cells present in cultures treated with cytokines alone (Figures 3B,C). These results suggest that VPA *ex vivo* expands phenotypically defined HSCs that possess low MMP and, hence, exhibit limited mitochondrial activity.

Since low MMP, which acts as a bioenergetic indicator of limited mitochondrial activity, has been reported to correlate with LT-repopulating potential of primary HSCs (Vannini et al., 2016; Liang et al., 2020), we sought to further address the relevance of mitochondrial activity in *ex vivo* expansion of HSCs with LT-reconstituting capacity. Thus, we assessed the MMP and correlated it with expression of HSC phenotypic markers. We found that the pool of VPA-expanded cells initiated with UCB-CD34^+^ cells was heterogeneous with regards to MMP (Figure 3D). This pool was comprised of two distinct subpopulations: the MMP-Low (TMRM-Low) and the MMP-High (TMRM-High). Expanded MMP-Low cells expressed significantly higher levels of CD34, CD90 and EPCR markers as opposed to MMP-High cells (Figures 3E–G). Importantly, this correlation persisted during the entire course of *ex vivo* expansion. We previously reported that VPA was also capable of expanding functional HSCs in *ex vivo* cultures initiated with either mobilized PB-CD34^+^ cells or BM-CD34^+^ cells. Similarly to expanded HSCs originated from UCB-CD34^+^ cells, expanded HSCs originated from PB- and BM-CD34^+^ cells established LT-engraftment and hematopoietic reconstitution in NSG mouse recipients (Zimran et al., 2020). Strikingly, expanded PB and BM HSCs with a CD34^+^CD90^+^ phenotype displayed reduced MMP compared to CD34^+^CD90^+^ cells present in cultures containing cytokine alone as determined by TMRM staining in either the presence or absence of verapamil (Zimran et al., 2020). It is important to emphasize that in cultures initiated with either UCB-, PB- or BM-CD34^+^ cells and treated with VPA, expanded HSCs that displayed low MMP did not undergo apoptosis, cell death or senescence, thus, excluding the possibility that the low MMP was a result of stress-induced cell death (Papa et al., 2018, 2019b; Zimran et al., 2020). To extend these observations, we next determined the correlation between MMP and expression levels of HSC phenotypic markers on HSCs present in VPA-expanded cultures initiated with either PB- or BM-CD34^+^ cells. Assessment of MMP in the presence of verapamil revealed that expanded PB-CD34^+^ cells as well as BM-CD34^+^ cells displayed low MMP and higher CD34 and CD90 expression levels as compared to cells with high MMP (Supplementary Figures 4A–F). The expanded CD34^+^CD90^+^ cells with high MMP expressed lower levels of CD34 and CD90 as opposed to expanded CD34^+^CD90^+^ cells with low MMP (Supplementary Figures 4G–L). Thus, regardless of the ontologic source of the CD34^+^ cells used to initiate the VPA-cultures, expanded cells were heterogeneous with regards to both their phenotypic and mitochondrial/metabolic status. Notably, these findings link the strong expression of HSC phenotypic markers to low mitochondrial activity.

### Single Expanded Cells Enriched for HSC Markers Possess Low MMP

**FIGURE 4:**
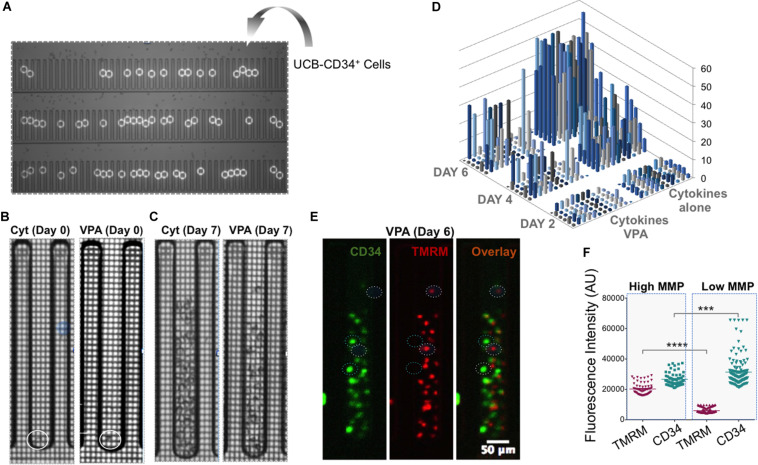
Single VPA-expanded cells enriched for HSC markers possess low MMP. **(A)** UCB-CD34^+^ cells were penned randomly as single cells using Opto-Electronic Positioning (OEP) force in wells of the nanofluidic BLI chip. White circles indicate location of single UCB-CD34^+^ cells at day 0. **(B,C)** Representative brighfield images of an individual well of BLI chip containing the culture initiating single UCB-CD34^+^ cell (**B**; circle indicates location of the single cell) and their progenies cultured with either cytokines (**C**; left panel) or VPA for 7 days (**C**; right panel). **(D)** The absolute number of cells (*z*-axis) present in individual wells of the BLI chip seeded with a single UCB-CD34^+^ cell and treated with either cytokine cocktail alone or with VPA for the indicated time points (Day 2, Day 4, and Day 6) as enumerated by an automated edge detection algorithm followed by visual confirmation of cells. **(E)** Representative fluorescent images of cells in a single well of the BLI chip cultured in the presence of VPA for 6 days (same well as shown in **C**). Left hand panel shows CD34 expression, middle panel shows TMRM expression and right hand panel shows the overlay. Circles denote cells that express high levels of CD34 and are not or slightly stained by TMRM or cells that do not or express very low CD34 levels and are strongly stained by TMRM. **(F)** Fluorescent intensities of TMRM and CD34 in single cells were determined using Fiji software and the “Measure” function with a multi-point selection of all individual cells in each of the pens that contain a greater number than 25 cells after the culture period. Error bars, SEM; ****p ≤ 0.0001; ***p < 0.001 as determined by unpaired *t*-test (two-tailed).

**FIGURE 5:**
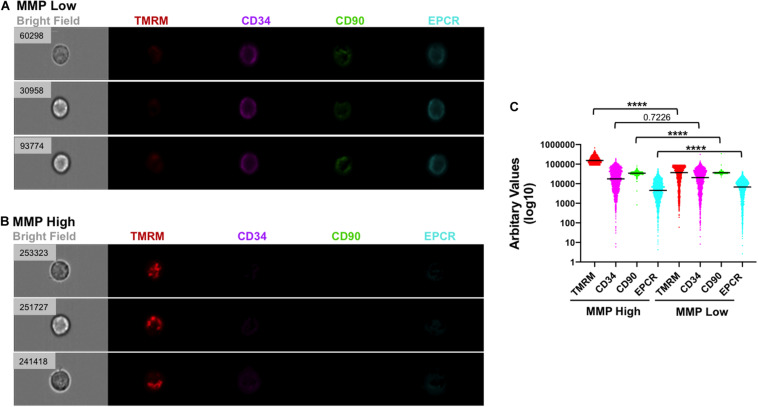
Low MMP correlates with strong expression of HSC markers in individual cells following VPA treatment. **(A,B)** Representative images of single cells generated in cultures treated with VPA for 4 days and captured using ImageStreamX with a 40× magnitude objective. Cells with low **(A)** and strong **(B)** staining for TMRM in the presence of verapamil were analyzed for expression of CD34 (purple), CD90 (green) and EPCR (light blue) using INSPIRE^®^ software. Numbers on the brightfield images represent the intensity of TMRM (arbitrary unit) staining of each indicated individual cell. **(C)** The intensity of TMRM, CD34, CD90, and EPCR staining across all VPA-expanded cells and clustered in MMP-High and MMP-Low groups according to TMRM staining intensity in the presence of verapamil. Median intensities in MMP-High and MMP -Low groups, were 154,543 and 36,778 for TMRM, 17,720 and 20,662 for CD34, 34,496 and 36,437 for CD90, and 4,524 and 6,817 for EPCR, respectively. ****p ≤ 0.0001 as determined by unpaired *t*-test (Mann–Whitney; two-tailed).

To determine whether the observed phenotypic and mitochondrial/metabolic heterogeneity of HSCs expanded with VPA was due merely to the heterogeneity of initiating UCB-CD34^+^ cells with various levels of CD34 expression or was also a consequence of VPA treatment, we utilized single cell platform nanofluidic chips equipped with Beacon OptoSelect technology (BLI). Specifically, single UCB-CD34^+^ cells were penned randomly and loaded into each individual well of the BLI nanofluidic chip using OEP force that was produced by a waveform generator (Figure 4A). Images of the culture wells was taken right after cell loading (day 0) and every 2 h thereafter over the entire 7-day culture period with either cytokines alone or VPA-containing media (Figures 4B,C, Supplementary Figures 5A,B, and Supplementary Videos). As anticipated, the number of viable expanded cells in wells of the nanofluidic chips that were treated with VPA-containing media was lower than the number of cells expanded in the wells of the nanofluidic chips treated with cytokines alone (Figure 4D). However, the number of expanded cells varied drastically among the wells of the nanofluidic chips containing media supplemented with VPA. We next assessed the MMP and expression of CD34 in expanded cells within individual wells initiated with a single UCB-CD34^+^ cell. Fluorescent microscopic analysis of the cells revealed that VPA-expanded cells within each individual well of the Beacon platform displayed varying degrees of TMRM and CD34 staining (Figure 4E). Notably, each expanded cell progeny originating from a single UCB-CD34^+^ cell that displayed robust CD34 staining exhibited low TMRM staining and vice-versa (Figure 4E). Thus, although starting with a single UCB-CD34^+^ cell, VPA treatment resulted in expansion of a heterogeneous population. Analyses of the entire population of expanded cells in all the wells that contained at least 25 cells confirmed a strong correlation between high CD34 expression and low TMRM staining (Figure 4F). In addition, we used ImageStream to assess and visualize expression of CD34, CD90 and EPCR in single VPA-expanded cells isolated based on the retention of TMRM staining in the presence of verapamil. A total of 1,620 live cells with low MMP and 1,619 cells with high MMP were analyzed. This high-resolution analysis revealed that MMP-low cells expressed significantly higher levels of CD90 and EPCR than the MMP-high cells (Figures 5A,B). Although it did not reach statistical significance, CD34 expression was higher in MMP-low cells (with median values of 20,652) as opposed to MMP-high cells (with median values of 17,720) (Figure 5C). Collectively, these data obtained by multiple classical and novel approaches indicate that VPA treatment also results in expansion of a phenotypic and mitochondrial/metabolic heterogeneous pool of HSCs. Remarkably, this expanded pool of HSCs is highly enriched for cells that are marked by high expression levels of phenotypic HSC markers and low mitochondrial activity, all properties that define unmanipulated HSCs with LT-repopulating potential.

### *Ex vivo*-Generated CD34^+^CD90^+^EPCR^+^ Cells With Low MMP Are Enriched for HSCs With LT-Functionality *in vitro*

**FIGURE 6:**
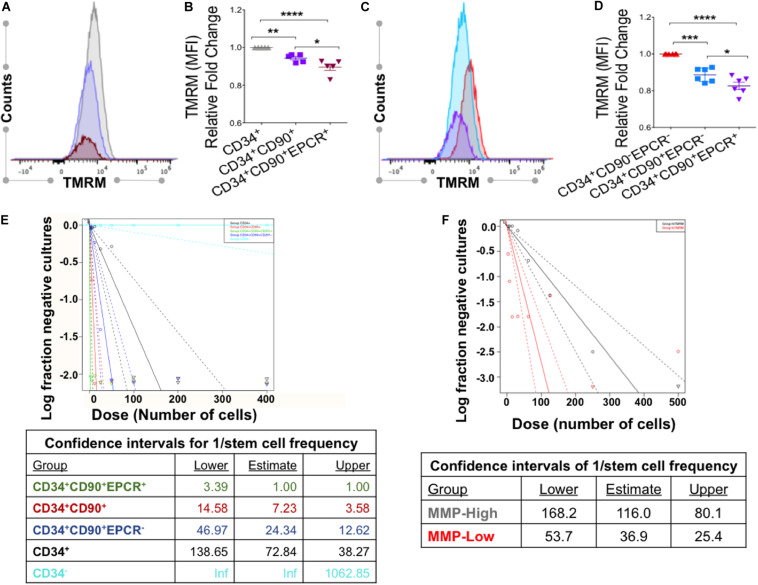
VPA-expanded cells with CD34^+^CD90^+^EPCR^+^ phenotype and low MMP display the greatest LTC-IC frequency. **(A)** Representative flow cytometry plot of TMRM staining, performed in the presence of verapamil, in CD34^+^ (gray), CD34^+^ CD90^+^ (purple), and CD34^+^CD90^+^EPCR^+^ (red) cells present in cultures treated with VPA for 4 days. **(B)** Relative fold change of median fluorescent intensity (MFI) in TMRM staining of the indicated cell populations evaluated as described in **(A)**. **(C)** Representative flow cytometry plot of levels of TMRM staining, performed in the presence of verapamil, in CD34^+^CD90^–^EPCR^–^ (red), CD34^+^CD90^+^EPCR^–^ (blue), and CD34^+^CD90^+^EPCR^+^ (purple) cells present in cultures treated with VPA for 4 days. **(D)** Relative fold change of median fluorescence intensity (MFI) of TMRM staining in the indicated cell populations measured as described in **(C)**. Fold-change is shown relative to CD34^+^ cells (C) and CD34^+^CD90^–^EPCR^–^ cells **(D)**. Error bars, SEM; *p ≤ 0.05; **p ≤ 0.01; ***p ≤ 0.001; ****p ≤ 0.0001 as determined by ordinary one-way ANOVA (Dunnett’s multiple comparison test). **(E)** Limiting dilution long-term culture-initiating cell (LTC-IC) assay and determination of stem cell frequencies (F) of various phenotypic cell subpopulation present in *ex vivo* cultures treated with VPA for 7 days. **(F)** Limiting dilution long-term culture-initiating cell (LTC-IC) assay and determination of stem cell frequencies at week 5 of MMP-high and MMP-low cells purified from day 7 *ex vivo* VPA-treated culture and distinguished based on TMRM retention in the presence of verapamil. *p*-value for the difference in stem cell frequencies between the MMP-High and MMP-Low groups is 2.44 × 10^–6^.

We next determined the MMP as well as mitochondrial mass and ROS levels in various subpopulations of phenotypically defined HSCs expanded with VPA. Flow cytometric analyses indicated that CD34^+^CD90^+^EPCR^+^ cells possess lower MMP, mitochondrial mass and ROS levels as compared to the CD34^+^CD90^+^ and CD34^+^ cell subpopulations (Figures 6A,B and Supplementary Figures 6A–C). In fact, these analyses revealed a strong inverse correlation between CD90 and EPCR expression, and MMP in VPA-expanded CD34^+^ cells in the presence of verapamil. Specifically, we found that cells expressing CD90 displayed a significantly reduced MMP as compared to CD34^+^ cells that lacked expression of CD90 (Figures 6C,D). This reduced MMP was even more pronounced in CD34^+^ cells expressing both CD90 and EPCR. Indeed, CD34^+^CD90^+^EPCR^+^ cells possessed a significantly lower MMP than CD34^+^CD90^+^EPCR^–^ cells. Of note, almost 99% of VPA-expanded cells with CD34^+^CD90^+^EPCR^+^ phenotype do neither express CD45RA nor CD38 (Supplementary Figures 7A–C). To test the functional capacity of VPA-expanded phenotypically defined HSCs, we performed the limiting dilution long-term culture-initiating cell (LTC-IC) assay, which is predictive of *in vivo* LT-functional potential. Cells lacking CD34 surface expression had zero LTC-IC potential. The frequency of LTC-IC potential was higher in those CD34^+^ cells that expressed CD90. Strikingly, the CD34^+^CD90^+^EPCR^+^ population had the highest LTC-IC frequency of all tested populations, including CD34^+^CD90^+^EPCR^–^ cells (Figure 6E). To assess and confirm that VPA-expanded cells with low MMP were more functionally fit than cells with a higher MMP profile, we next performed limiting dilution of LTC-IC assays with MMP-High and MMP-Low cells purified from VPA cultures irrespective of their phenotype. This analysis revealed that MMP-Low expanded cells possessed a significantly higher frequency of LTC-IC (*p*-value 2.44 × 10^–6)^ than MMP-High cells at week 5 (Figure 6F). A similar trend in stem cell frequency between MMP-High and MMP-Low groups was observed at week 7 (1 in 173.9 vs. 1 in 61.3, respectively; *p* = 2.95 × 10^–6^) (data not shown). Collectively, these data indicate that subpopulation of VPA-expanded CD34^+^CD90^+^EPCR^+^ cells, which possess the lowest mitochondrial activity is the most functionally fit among all the other phenotypically defined HSCs present in VPA-expanded grafts with LT-repopulating capacity in both murine models and human recipients.

## Discussion

The identification of the HSC phenotypic markers whose expression best correlate with the transcriptome and mitochondrial/metabolic profile that most closely resembles that of unmanipulated LT-HSCs is the key to identify LT-HSCs in *ex vivo* expansion cultures. In this study, we report that the pool of VPA-expanded HSCs, which are capable of establishing LT-hematopoiesis (Chaurasia et al., 2014), is comprised of cells marked by high expression (mRNA and protein) of CD34, CD90 and most importantly EPCR. Remarkably, the highest expression levels of these HSC phenotypic markers are tightly coupled to low MMP and, hence, mitochondrial activity that characterizes primary HSCs with LT-repopulating capacity. In fact, within the heterogeneous pool of VPA-expanded HSCs with demonstrated *in vivo* repopulating potential, the most functionally fit cells are those of the CD34^+^CD90^+^EPCR^+^ phenotype and low MMP.

Exposure of HSCs to cytokine supplemented cultures greatly compromises their LT-repopulating potential despite a dramatic increase in the number of immunophenotypically identified HSCs. Although a high number of phenotypically defined human HSCs is critical, it is not predictive of engraftment and hematopoietic reconstitution outcomes (Psatha et al., 2017). Indeed, the functional identity of *ex vivo* expanded HSCs has been frequently discordant with their phenotype. Beyond the phenotype, the transcriptome and the metabolic profile are essential determinants of the repopulating potential of *ex vivo* expanded HSCs and dictate their biological function.

We previously reported that *ex vivo* VPA-expanded human HSCs promote LT-reconstitution in serially transplanted murine recipients (Chaurasia et al., 2014). Notably, expanded HSCs marked by expression of CD34 and CD90 exhibit a restructured primitive mitochondrial network that is characterized by reduced mitochondrial mass, ROS and MMP compared to the same subpopulation of cells presents in cultures containing cytokines alone. VPA-expanded cells possess a rewired glycolytic landscape and rely more on glycolysis than on mitochondrial OXPHOS. Importantly, VPA-expanded HSCs display a transcriptomic signature that closely resembles that of unmanipulated LT-HSCs. Although VPA-expanded CD34^+^ cells are heterogeneous, they are, however, greatly enriched for gene repertories that not only mark but also govern quiescence, metabolism and LT-HSC behavior (Papa et al., 2018).

Besides VPA, several additional *ex vivo* expansion strategies, including those utilizing SR1, NAM and UM171 have recently shown encouraging results in pre-clinical studies and more importantly, in ongoing clinical trials (Wagner et al., 2016; Horwitz et al., 2019; Cohen et al., 2020). The infusion of these *ex vivo*-expanded grafts has led to rapid and sustained multilineage hematopoietic reconstitution in allogeneic HSC recipients. Notably, EPCR expression marks UM171-*ex vivo* expanded LT-HSCs. Originally described as a key component of endothelial barrier protection, EPCR has been reported to identify murine HSCs and regulate HSC retention in the BM (Balazs et al., 2006; Kent et al., 2009; Gur-Cohen et al., 2016). Consistent with these findings, genetically modified mice with low expression levels of EPRC are characterized by defects in HSC BM homing (Gur-Cohen et al., 2015; Fares et al., 2017). Remarkably, UM171-expanded human LT-HSCs that express high levels of EPCR are greatly enriched for CD90 expression (Fares et al., 2017). Both UM171 and SR1 strategies expand HSC grafts that establish LT-hematopoiesis, yet, they do not induce the same transcriptomic changes and same cell surface marker expression (Fares et al., 2014). In contrast to UM171, SR1 does neither induce expression of EPCR nor CD90 (Fares et al., 2014). In fact, SR1 treatment appears to target phenotypically less primitive cells and generates cells with less-durable self-renewal activity than those generated by UM171 treatment (Fares et al., 2014). Remarkably, the potency of SR1-expanded grafts has been reported to be relatively lower than that of grafts expanded with UM171 (Fares et al., 2014). Taking advantage of our VPA *ex vivo* expansion culture and utilizing both bulk RNA-seq as well as high-resolution single-cell RNA-seq, we demonstrated that VPA-expanded HSCs are marked by robust expression of HSC phenotypic markers. Among those markers, both CD90 and EPCR emerged as the most strongly induced by VPA, not only when compared to cultures treated with cytokines alone but importantly when compared to unmanipulated UCB-CD34^+^ cells. Notably, at single cell resolution, cells characterized by the strong expression of CD34, CD90 and EPCR display transcriptomic signatures that resemble that of unmanipulated human LT-HSCs. Moreover, VPA-expanded CD34^+^CD90^+^EPCR^+^cells contained the highest frequency of LTC-IC. These findings are consistent with our previous report that the pool of VPA-expanded HSCs possesses a transcriptomic landscape that is highly enriched for gene signatures that characterize unmanipulated human and non-human HSCs with LT-regenerative capacity (Ivanova et al., 2002; Novershtern et al., 2011; Gazit et al., 2013; Chen et al., 2014; Radtke et al., 2017; Papa et al., 2018).

In addition to the transcriptome, metabolism is another critical regulator of LT-repopulating capacity of primary HSCs. Both transcriptomic and metabolic properties are intrinsically coupled to mitochondrial activity, which is a central regulator of HSC activation, proliferation, differentiation and adaptation to stress, and thus, determines HSC fate decisions. Primary LT-HSCs have been reported to retain a primitive mitochondrial network that is characterized by low MMP and ROS generation (Jang and Sharkis, 2007; Mantel et al., 2015; Vannini et al., 2016; Papa et al., 2019b; Liang et al., 2020). Recently, this notion has been challenged however by new evidence indicating that HSCs exhibit higher MMP than the more committed multiprogenitor and hematopoietic cells (Bonora et al., 2018; Morganti et al., 2019). While these findings can be in part explained by HSC heterogeneity, HSCs have been shown to exhibit a high xenobiotic pump activity, which extrudes mitochondrial dyes and, therefore, influences precise MMP measurement.

We previously demonstrated that human HSCs generated in VPA containing *ex vivo* cultures possess lower mitochondrial activity, and specifically MMP and ROS production than HSCs of the same phenotype present in cultures containing cytokines alone (Papa et al., 2018; Zimran et al., 2020). In marked contrast with cytokine-expanded grafts, VPA-expanded grafts are capable of establishing LT-sustained multilineage hematopoietic reconstitution (Chaurasia et al., 2014; Zimran et al., 2020). Given the new understanding of the importance of the efflux pumps, we assessed MMP of VPA-expanded HSCs in the presence of the pump inhibitor, verapamil. Consistent with our previous evidence, expanded HSCs in VPA cultures initiated with CD34^+^ cells isolated from various sources including UCB, BM or mobilized PB exhibited a lower MMP than those present in cytokine cultures even in the presence of verapamil. While in this study, we do not compare human expanded HSCs with other cell subpopulations including committed progenitors, our results are in agreement with reports that low mitochondrial activity and MMP identifies unmanipulated, rare murine HSCs with LT-self renewal capacity (Vannini et al., 2016). Indeed, a recent report revealed that low MMP marks label retention defined dormant murine LT-HSCs (Liang et al., 2020).

Emerging evidence has emphasized that phenotype does not always recapitulate stem cell function of *ex vivo* expanded HSCs (Psatha et al., 2017; Chen et al., 2019). In agreement with these reports, our findings raise the possibility that despite an HSC phenotype, expanded cells displaying high MMP and lower levels of HSC phenotypic markers might be primed to commitment and differentiation, and, thus, might not reflect HSCs with LT-reconstituting ability. Certainly, serial transplantation of *ex vivo* expanded human CD34^+^CD90^+^EPCR^+^ cells with strong expression of these phenotypic markers and low MMP would confirm their *in vivo* LT-regenerative capacity.

Together with our previously published evidence (Papa et al., 2018), our findings indicate that acquisition of an HSC transcriptome associated with strong expression of HSC phenotypic markers is profoundly coupled to a remodeled primitive mitochondrial network that exhibits low MMP as well as low mitochondrial mass and ROS generation. In fact, as previously shown, a further decrease in MMP levels under hypoxic condition resulted in a significant increase in the percentage of VPA-expanded HSCs (Papa et al., 2018) emphasizing that MMP and, hence, limited mitochondrial activity is an essential property of human LT-HSCs. Yet, the molecular mechanisms underlying such a restructured mitochondrial network, which is characterized by low mitochondrial activity, remain unknown. Whether the remodeled mitochondrial network and increased glycolysis is a consequence of VPA-induced stemness or vice-versa is also unknown. It is also tempting to speculate that both stemness and remodeling of the mitochondrial network occur simultaneously and dynamically regulate each other to establish cellular reprogramming and *ex vivo* expansion of HSCs with LT hematopoietic reconstitution. Indeed, these are the subject of our ongoing investigations at single cell resolution, which aim to uncover the molecular programs and mechanisms that coordinate these events.

Collectively, our findings indicate that reduced mitochondrial activity is restricted to VPA-expanded cells with the highest transcriptomic and protein expression of CD34, CD90 and EPCR phenotypic markers. Together, these properties can be used to identify human functional LT-HSCs in *ex vivo* expansion cultures. Moreover, such an approach that integrates both expression levels of phenotypic markers and low MMP will facilitate development of a potency assay that can be used to quantify the number of LT-HSCs not only following *ex vivo* expansion but also *ex vivo* genetic modification of human HSCs for therapeutic purposes.

## Data Availability Statement

The bulk mRNA-seq data for Day 0, Day 2, and Day 4 are available at Gene Expression Omnibus (GEO) under GSE110968 whereas the bulk RNA-seq files for Day 6 are available under GSE155180. The single-cell RNA-seq data for Day 0 and 2 are available under GSE110973.

## Author Contributions

LP conceived, designed, performed the experiments, supervised the study, analyzed the data, and wrote the manuscript. MD performed the experiments and analyzed the data. TM performed the bulk and single cell analyses and assisted with manuscript preparation. MZ performed the experiments and analyzed the data. KB performed the experiments, image processing, and analyses of single cells on the BLI platform. RS supervised the BLI single cell experiments, single-cell RNA-seq assay, and data analyses. RP supervised the bulk and single-cell gene expression analyses. CS designed, performed the experiments, supervised the study, analyzed the data, and wrote the manuscript. RH supervised the study, wrote and approved the manuscript, and provided the financial support. All the authors contributed to the article and approved the submitted version.

## Conflict of Interest

The authors declare that the research was conducted in the absence of any commercial or financial relationships that could be construed as a potential conflict of interest.
