# Supplementary material for: Limited Mitochondrial Activity Coupled With Strong Expression of CD34, CD90 and EPCR Determines the Functional Fitness of ex vivo Expanded Human Hematopoietic Stem Cells
Source: Front Cell Dev Biol. 2020 Dec 15;8:592348. doi: 10.3389/fcell.2020.592348 (PMC7769876; doi:10.3389/fcell.2020.592348)
Supplement: Supplementary file 1 [file Table_1.DOCX]

**SUPPLEMENTARY FIGURE LEGENDS**

**Figure S1. VPA treatment generates ex vivo CD34^+^C90^+^CD38^-^CD45RA^-^ cells. (A, B)** Percentage (A) and absolute number (B) of viable HSCs of the CD34^+^CD90^+^CD38^-^CD45RA^-^ phenotype generated throughout 6 days of culture of UCB-CD34^+^cells with either cytokines alone or in combination with VPA (Cyt+VPA) as measured by acridine orange/propidium iodide staining and assessed by flow cytometry analysis (n=3). Error bars, SEM; ****, p≤0.0001; **, p≤0.01 as determined by 2-way ANOVA (Sidak’ multiple comparison test).

**Figure S2. VPA-expanded cells possess a distinct transcriptomic profile. (A, B)** Volcano plot indicating differentially expressed genes between CD34^+^ cells isolated from the 2-day (A) or from the 6-day (B) cultures treated with VPA or cytokines alone and analyzed by bulk RNA-seq (n=3). A total of genes in (A) and 30,456 with a fold-change (FC) cut off of 2 and p-value cutoff of 8x10^-4^ were characterized. Blue dots represent significantly down-regulated genes with a Log_2_ fold change >2 in VPA-CD34^+^cell cultures compared to those in cultured cells treated with cytokines alone. Yellow dots represent significantly up-regulated genes with a Log_2_ fold change >2 in VPA- CD34^+^cell cultures compared to those cultured with cytokines alone. The transcripts for HSC phenotypic markers EPCR, CD90 and CD49f are highlighted. **(C)** Heatmap of glycolysis hallmarks in cultures of CD34^+^ cells treated with cytokines alone (gray) and cultures of CD34^+^ cells treated with VPA (red) for 4 days.

**Figure S3. Electron transport genes in VPA-expanded CD34^+^cells.** Heatmap of mitochondrial electron transport chain genes in 9 transcriptionally different clusters of CD34^+^ cells expanded with VPA for 2 days analyzed by single cell RNA-seq (WP gene sets). These cells are also clustered based on LT-HSC score based on correlation with Ivanova gene sets specific to human LT-HSCs.

**Figure S4. VPA-generated cells in ex vivo cultures initiated with PB- or BM-CD34^+^ cells that exhibit low MMP express high levels of the HSC phenotypic markers. (A)** Representative flow cytometry plot of TMRM staining performed in the presence of verapamil in cells generated in cultures initiated with PB-CD34^+^cells and treated with VPA for 7 days. **(B, C)** 25 % of VPA-expanded cells strongly and 25% of cells slightly stained with TMRM in A were further analyzed by flow cytometry in B and C for expression levels of CD34 and CD90, respectively. **(D)** Representative flow cytometry plot of TMRM staining performed in the presence of verapamil in cells generated in cultures initiated with BM-CD34^+^cells and treated with VPA for 7 days. **(E, F)** 25 % of VPA-expanded cells strongly or slightly stained with TMRM in D were further analyzed by flow cytometry for expression levels of CD34 (E) and CD90 (F), respectively. **(G)** Representative flow cytometry plot of TMRM staining performed in the presence of verapamil, in CD34^+^CD90^+^ cells generated in cultures initiated with PB-CD34^+^cells and treated with VPA for 7 days. **(H, I)** 25 % of VPA-expanded cells strongly or slightly stained with TMRM in G were further analyzed by flow cytometry for expression levels of CD34 (H) and CD90 (I), respectively. **(J)** Representative flow cytometry plot of TMRM staining performed in the presence of verapamil in CD34^+^CD90^+^ cells generated in cultures initiated with BM-CD34^+^ cells and treated with VPA for 7 days. **(K, L)** 25 % of VPA-expanded cells strongly or slightly stained with TMRM in J were further analyzed by flow cytometry for expression levels of CD34 (K) and CD90 (L), respectively (n=2). Numbers represent the average of median florescence intensity (MFI) of cells that retain low TMRM staining and cells that retain high TMRM staining.

**Figure S5. VPA-generated cells in BLI platforms are viable. (A)** Brightfield images of cell growth in BLI platform after 7 days of culture with VPA. **(B)** Viability of cells cultured in the BLI platform treated for 7 days with VPA and stained with Calcein, AM. Images were captured on the FITC channel.

**Figure S6. VPA-expanded cells with CD34^+^CD90^+^EPCR^+^ phenotype generate low mitochondrial ROS levels and exhibit a low mitochondrial mass. (A)** Representative flow cytometry histograms of mitochondrial ROS levels in the indicated subsets (red: CD34^+^CD90^+^EPCR^+^; blue: CD34^+^CD90^+^; gray: CD34^+^) of VPA-expanded cells for 4 days as determined by staining with MitoSOX*™*Red dye. **(B)**. Fold change of ROS levels (MFI) in CD34^+^CD90^+^EPCR^+^ and CD34^+^CD90^+^ cells relative to CD34^+^ cells measured as described in A (n=2). **(C)** Representative flow cytometry histograms of mitochondrial mass in the indicated subsets (red: CD34^+^CD90^+^EPCR^+^; blue: CD34^+^CD90^+^; gray: CD34^+^) of VPA-expanded cells for 4 days as determined by staining with MitoTrackerGreen^FM^ dye in the presence of verapamil (n=2). **(D)**. Fold change of mitochondrial mass (MFI) in CD34^+^CD90^+^EPCR^+^ and CD34^+^CD90^+^ cells relative to CD34^+^ cells measured as described in C.

**Figure S7. VPA-generated cells with CD34^+^CD90^+^EPCR^+^ phenotype lack expression of CD38 and CD45RA. (A)** Representative flow cytometry plot of VPA-expanded cells expressing CD34 in cultures generated with VPA treatment for 7 days. **(B)** Representative flow cytometry plot of VPA-expanded CD34^+^ cells generated in cultures treated with VPA for 7 days evaluated for expression of CD90A and EPCR. **(C)** Representative flow cytometry plot of VPA-expanded CD34^+^CD90^+^EPCR^+^ cells in cultures generated with VPA treatment for 7 days evaluated for expression of CD38 and CD45RA.

**Videos:**

Video 1: Movie of UCB-CD34^+^ cells during random single cell penning in individual wells of the BLI nanofluidic chip using Beacon Opto-Electronic Positioning force. White circles indicate location of single UCB-CD34^+^ cells at day 0.

Video 2: Movie of cell growth in one representative well of the BLI nanofluidic chip over the 7-day of culture period in media supplemented with cytokine cocktail alone.

Video 3: Movie of cell growth in one representative well of the BLI nanofluidic chip over the 7-day of culture period in media supplemented with the combination of cytokines and VPA.
