# Supplementary figures and images for: Limited Mitochondrial Activity Coupled With Strong Expression of CD34, CD90 and EPCR Determines the Functional Fitness of ex vivo Expanded Human Hematopoietic Stem Cells
Source: Front Cell Dev Biol. 2020 Dec 15;8:592348. doi: 10.3389/fcell.2020.592348 (PMC7769876; doi:10.3389/fcell.2020.592348)

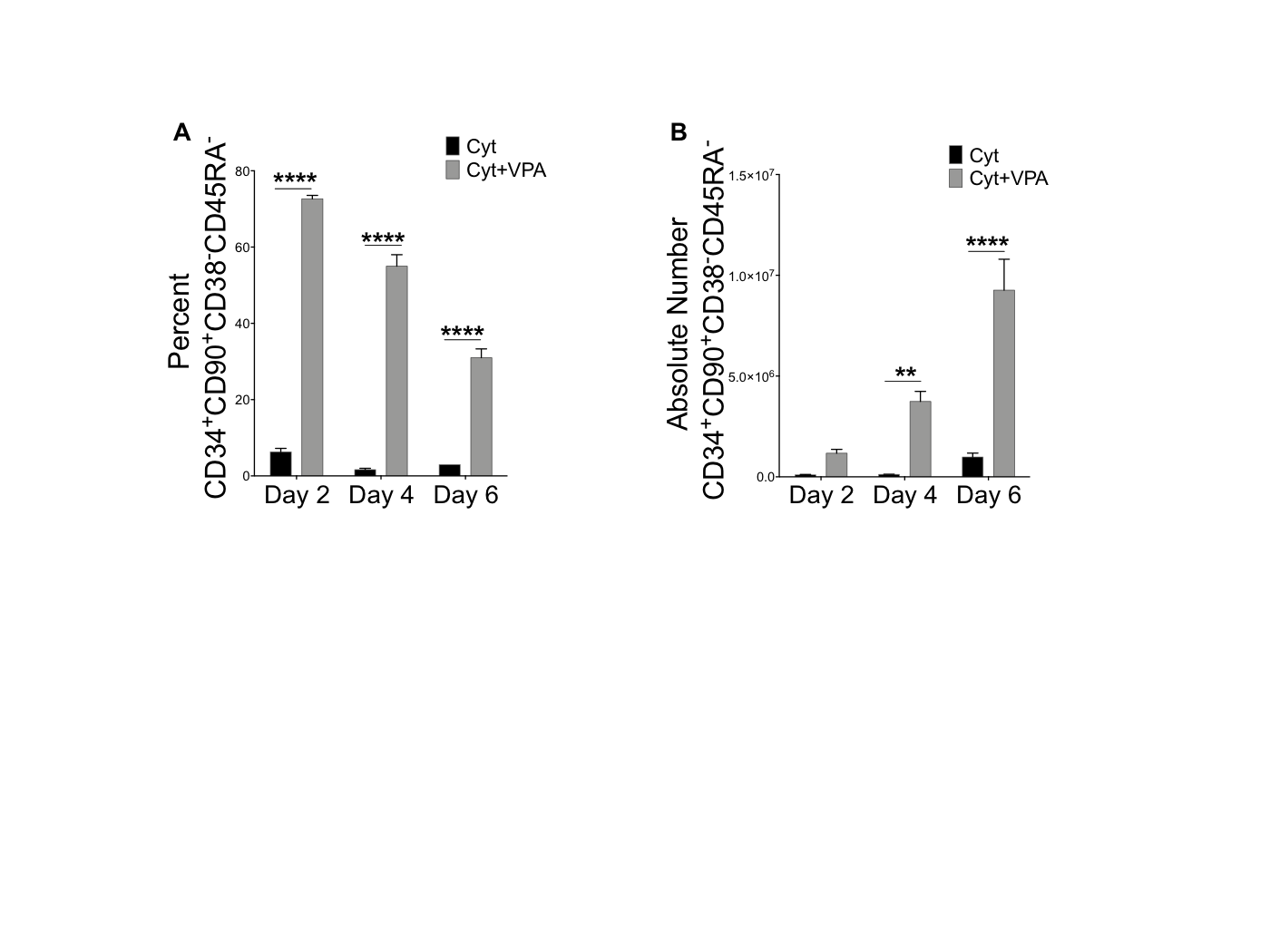

Supplement: Supplementary file 5 [file Image_1.TIFF]

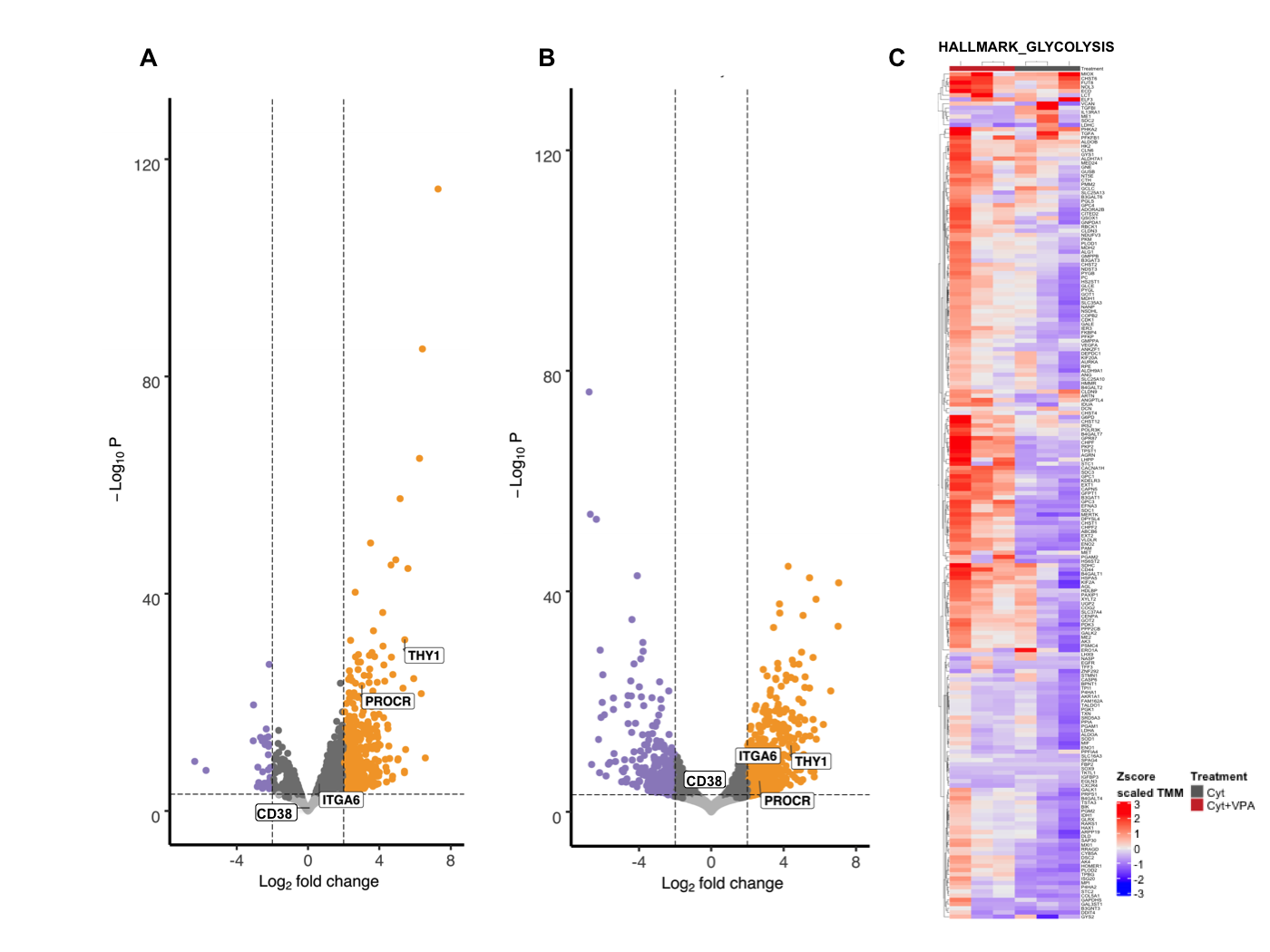

Supplement: Supplementary file 6 [file Image_2.TIFF]

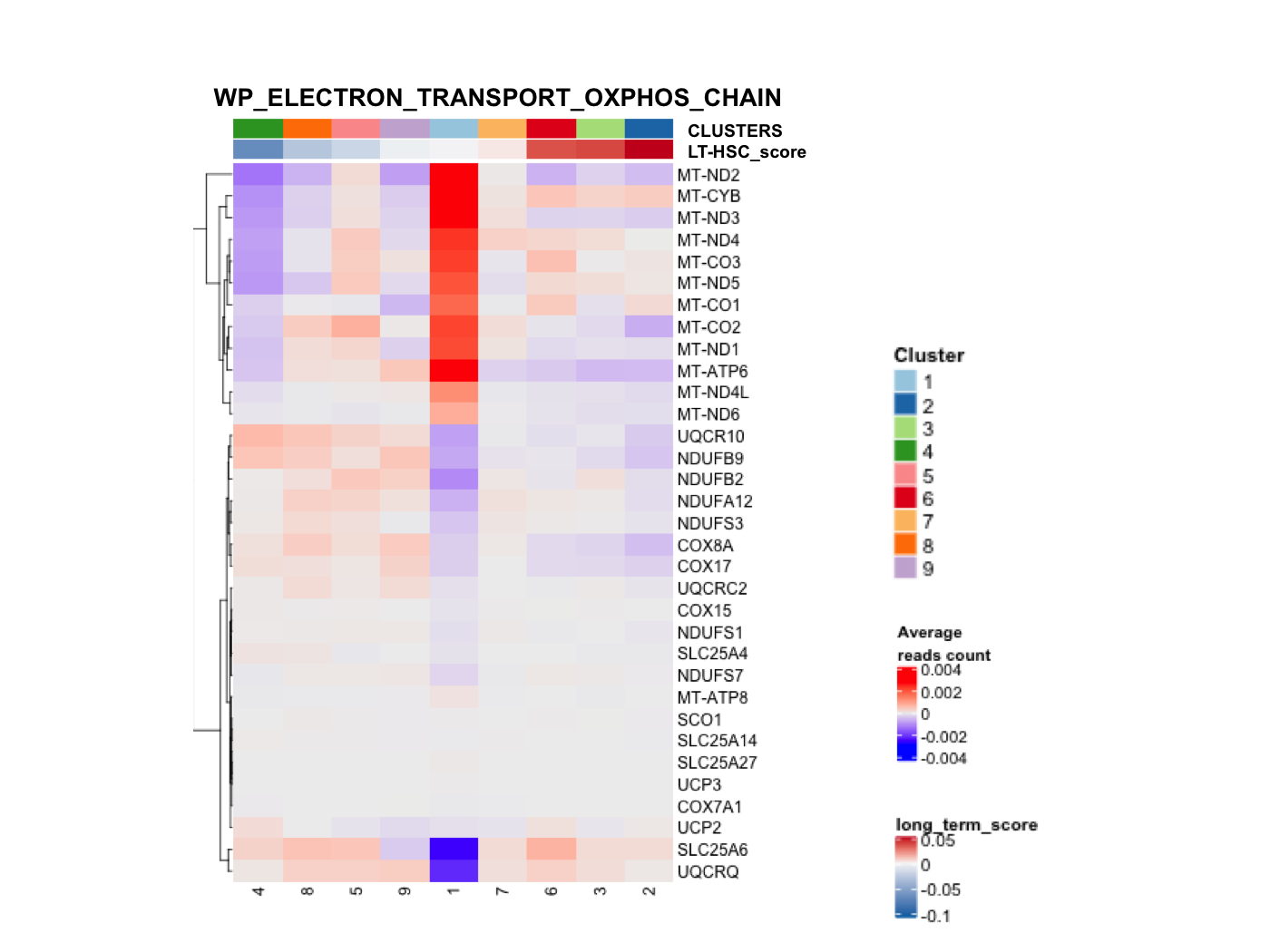

Supplement: Supplementary file 7 [file Image_3.tiff]

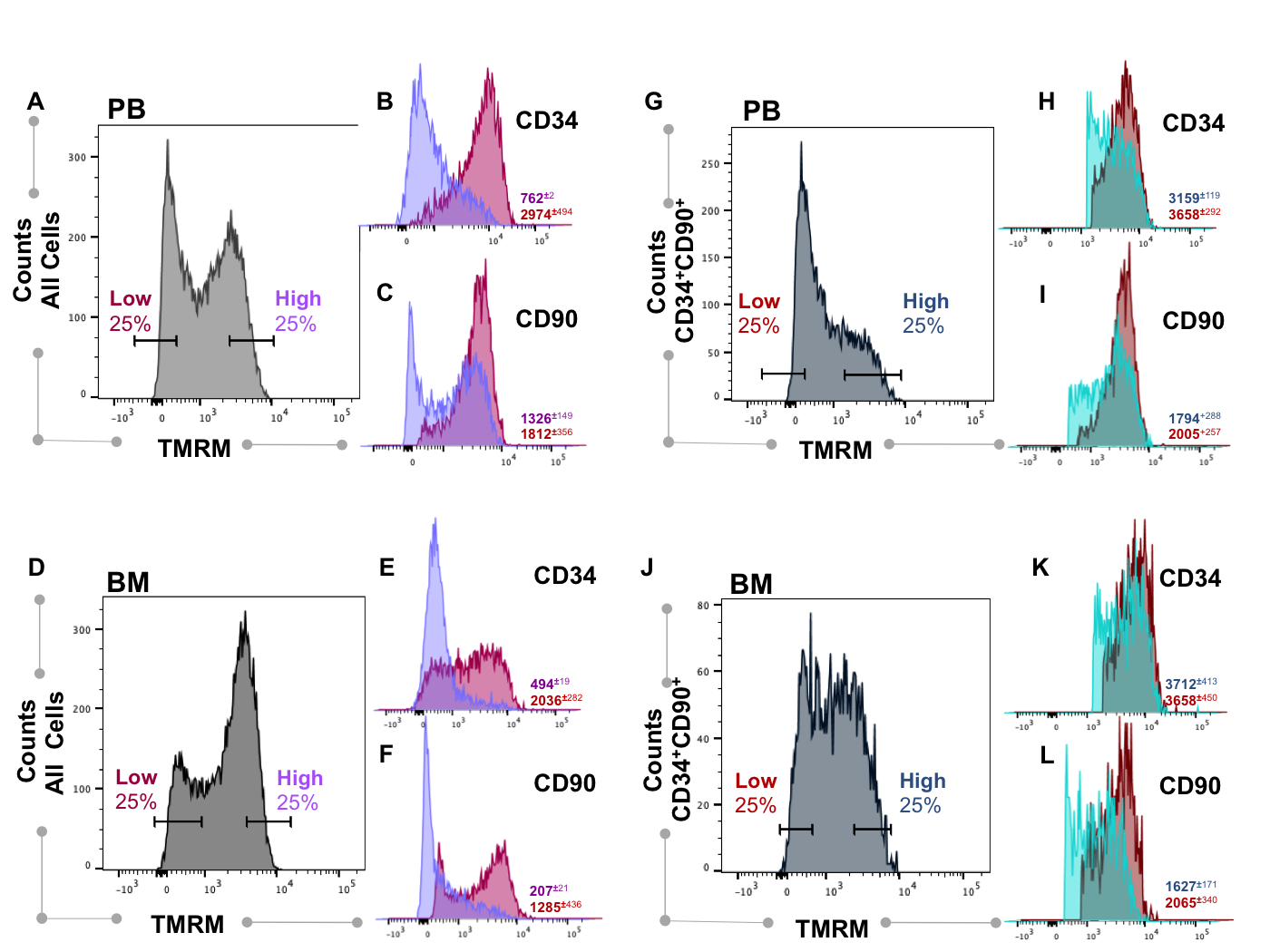

Supplement: Supplementary file 8 [file Image_4.TIFF]

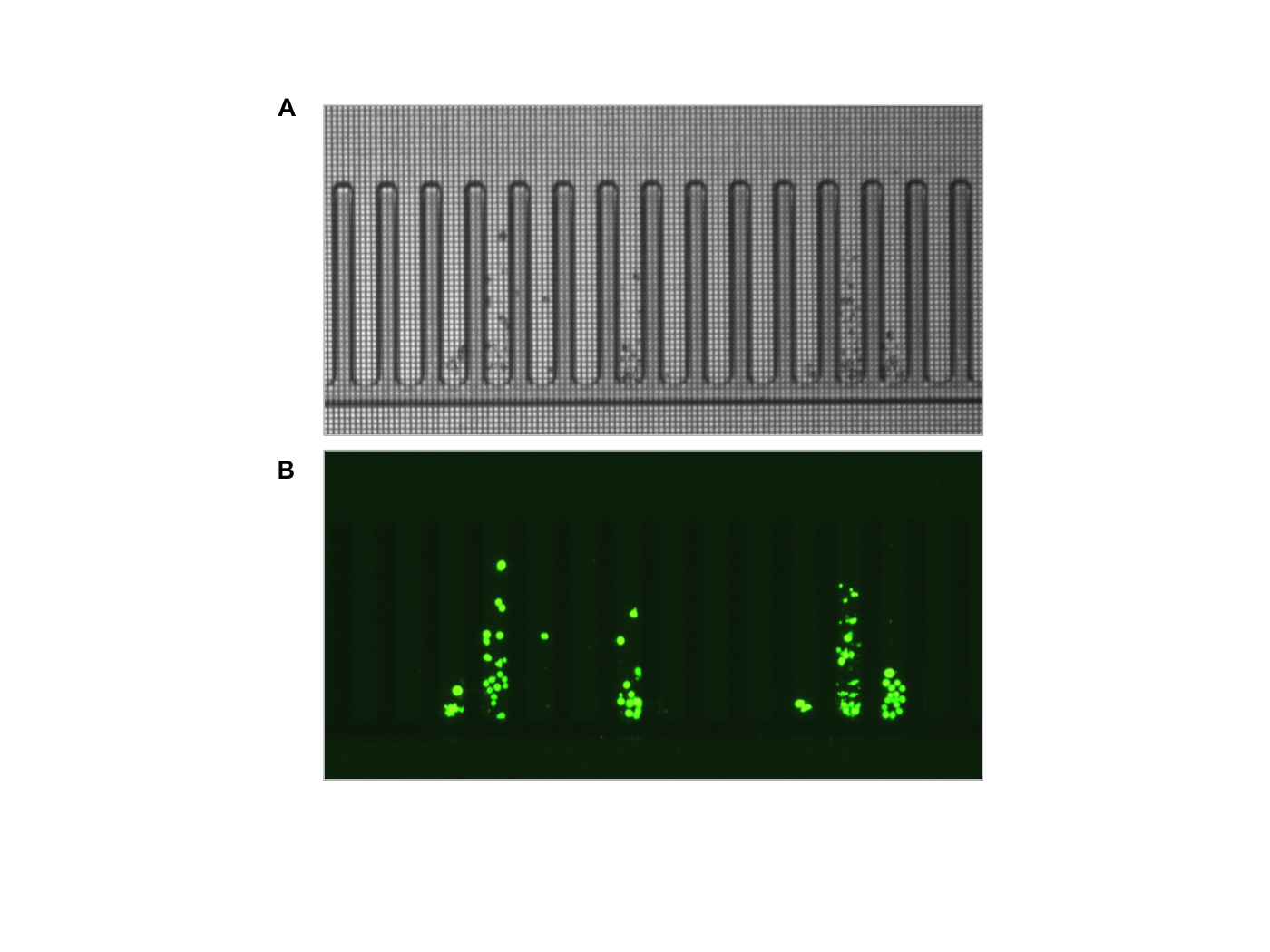

Supplement: Supplementary file 9 [file Image_5.TIFF]

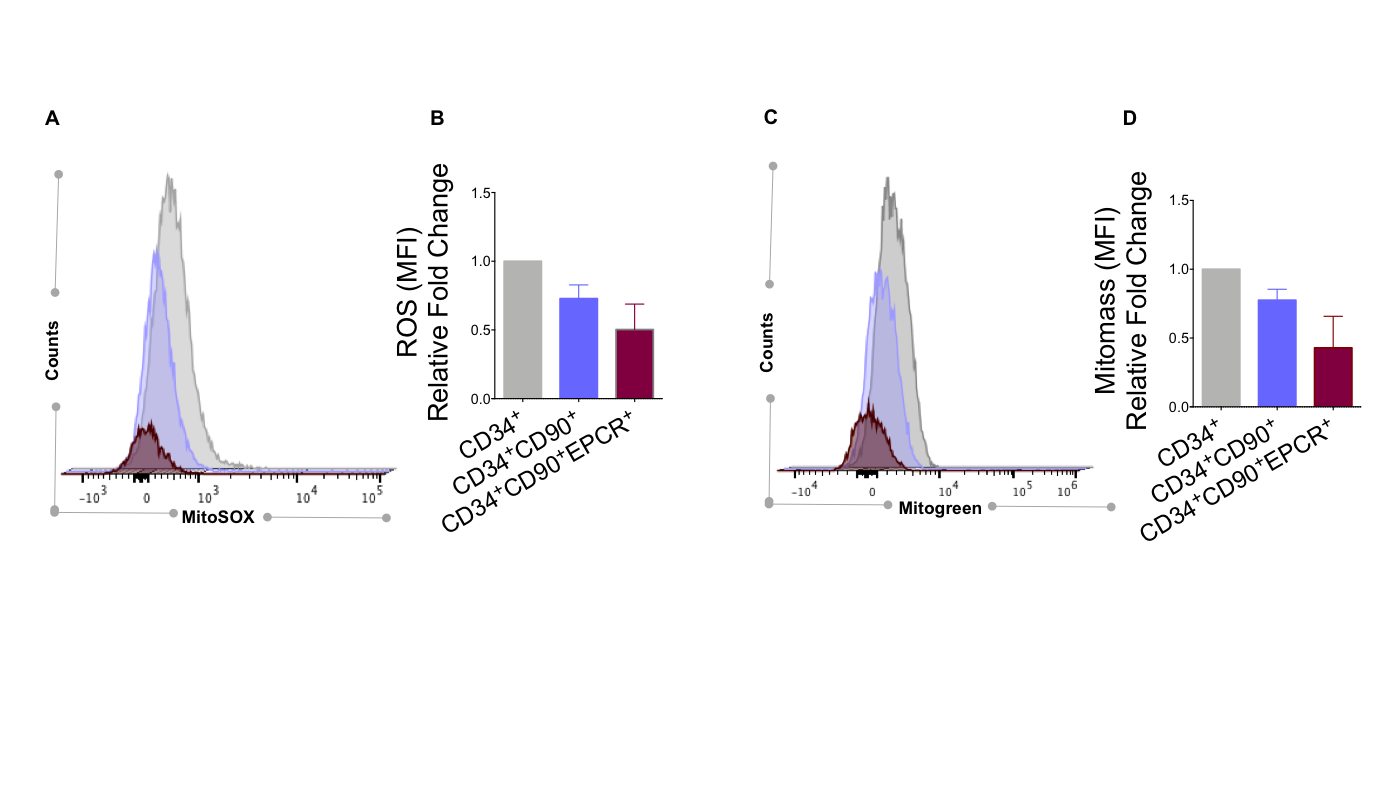

Supplement: Supplementary file 10 [file Image_6.TIFF]

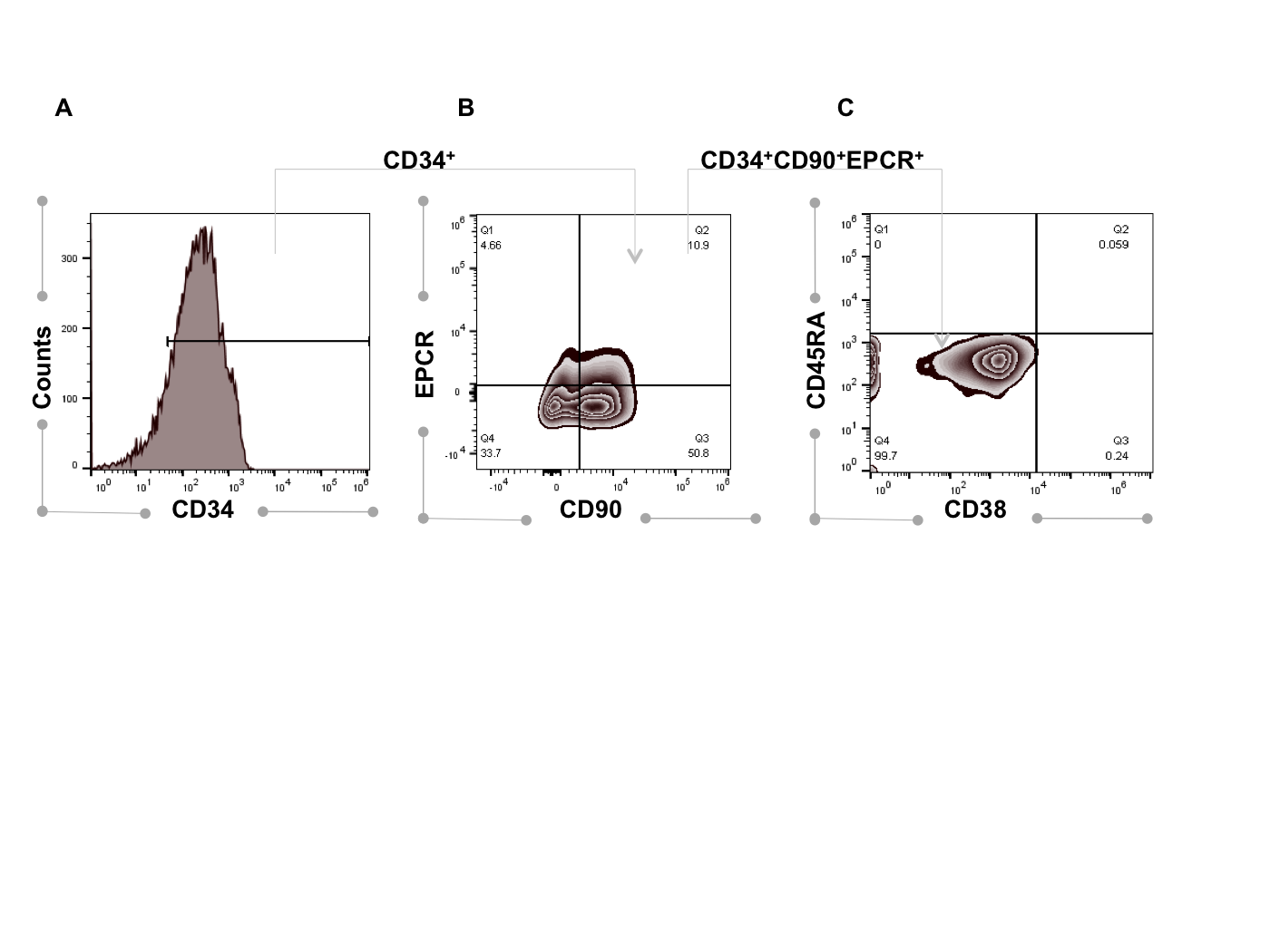

Supplement: Supplementary file 11 [file Image_7.TIFF]
